# Bragg coherent diffraction imaging of single 20 nm Pt particles at the ID01-EBS beamline of ESRF

**DOI:** 10.1107/S1600576722002886

**Published:** 2022-05-16

**Authors:** M.-I. Richard, S. Labat, M. Dupraz, N. Li, E. Bellec, P. Boesecke, H. Djazouli, J. Eymery, O. Thomas, T. U. Schülli, M. K. Santala, S. J. Leake

**Affiliations:** a Université Grenoble Alpes, CEA Grenoble, IRIG, MEM, NRS, 17 rue des Martyrs, 38000 Grenoble, France; b ESRF – The European Synchrotron, 71 Avenue des Martyrs, 38000 Grenoble, France; c Aix Marseille Université, CNRS, Université de Toulon, IM2NP UMR 7334, 13397 Marseille, France; d Department of Mechanical, Industrial and Manufacturing Engineering, Oregon State University, Corvallis, Oregon, USA

**Keywords:** Bragg coherent diffraction imaging, Extremely Brilliant Source, structure, Pt particles, three-dimensional, nanoscale

## Abstract

Electronic or catalytic properties can be modified at the nanoscale level. Engineering efficient and specific nanomaterials requires the ability to study their complex structure–property relationships. Here, Bragg coherent diffraction imaging was used to measure the three-dimensional shape and strain of platinum nanoparticles with a diameter smaller than 30 nm, *i.e.* significantly smaller than any previous study. This was made possible by the realization of the Extremely Brilliant Source of ESRF, The European Synchrotron. This work demonstrates the feasibility of imaging the complex structure of very small particles in three dimensions and paves the way towards the observation of realistic catalytic particles.

## Abstract 2

This work demonstrates three-dimensional Bragg coherent diffraction imaging of single 20 nm Pt particles at the ID01-EBS beamline of ESRF.

## 1. Introduction

Nanoparticles are used as catalysts for a wide range of chemical reactions. Correlating how parameters that influence catalyst performance, *i.e.* nanoparticle size, shape, strain, redox functionality and metal–support interactions, affect and evolve in core catalytic processes is mandatory for improving industrial processes. Recent experiments have shown that Bragg coherent diffraction imaging (BCDI) is a powerful tool in this regard (Ulvestad *et al.*, 2016 ▸; Ulvestad & Yau, 2017 ▸; Kim *et al.*, 2018 ▸; Fernández *et al.*, 2019 ▸; Abuin *et al.*, 2019 ▸; Carnis, Kshirsagar *et al.*, 2021 ▸). The technique provides the crystal’s shape as well as three-dimensional displacement and hence strain fields from which surface reactivity maps can be inferred (Watari *et al.*, 2011 ▸). However, all experiments published so far have investigated particles larger (≥60 nm) than those used in practical applications. For instance, quantum confinement effects begin to occur around 5–30 nm (Li *et al.*, 2006 ▸; Pauzauskie & Yang, 2006 ▸) and the typical size of nanocatalysts is often smaller than 30 nm (Long *et al.*, 2013 ▸). Materials have a higher specific surface when their particle size is reduced. As an example, a nanoparticle with a diameter of 30 nm has ∼5% of its atoms on the surface, while a particle of 3 nm has ∼50% of its atoms on the surface. The particle-size reduction is also often assisted by an enrichment in surface structures (crystal edges, corners and faces), which results in a higher efficiency of the catalysts. Recently, Björling and co-workers demonstrated the possibility to measure 60 nm Au nanoparticles (Björling *et al.*, 2019 ▸). They succeeded in measuring and reconstructing two-dimensional projection images as well as three-dimensional data sets from particles undergoing uncontrolled and unknown rotations (Björling *et al.*, 2020 ▸).

The main challenges in investigating smaller particles are (i) the weak scattering signal which rapidly decreases with the particle size, (ii) the need for stabilizing the nanoparticles over the timescale of the experiment and under the intense beam, and (iii) the mechanical/thermal drift of the sample as well as the eccentricity or wobble of the high-resolution diffractometer during data collection, *i.e.* the rocking curve (rotation of the crystal around a single axis by several degrees). Work on mitigating the angular uncertainties in BCDI has been recently published (Calvo-Almazán *et al.*, 2019 ▸; Björling *et al.*, 2019 ▸). For a perfect crystal, the maximum of the scattered intensity is proportional to the square of the particle volume, the total diffracted intensity scales with particle size *d* as *d* ^3^ and the integrated intensity of the central two-dimensional slice scales as *d* ^4^. A decrease of one order of magnitude of the diameter of a spherical particle leads to a decrease of six orders of magnitude of its maximum scattered intensity, three orders of magnitude of the total diffracted intensity and four orders of magnitude of the integrated intensity of the central two-dimensional slice.

The new Extremely Brilliant Source (EBS) (Raimondi, 2016 ▸) of ESRF, The European Synchrotron, provides unprecedented coherent flux densities and therefore has the potential to produce high-resolution coherent diffraction patterns from very small crystals. Here, we demonstrate the ability to measure nanoparticles as small as 20 nm with the new capabilities offered by the EBS. To overcome sample instabilities, we focused on platinum (Pt) nanoparticles embedded in sapphire. They are stable under the X-ray beam. We also demonstrate that it is feasible to image three-dimensional particles close to the relevant size for *e.g.* optoelectronic or catalytic applications.

## 2. Materials and methods

**Figure 1:**
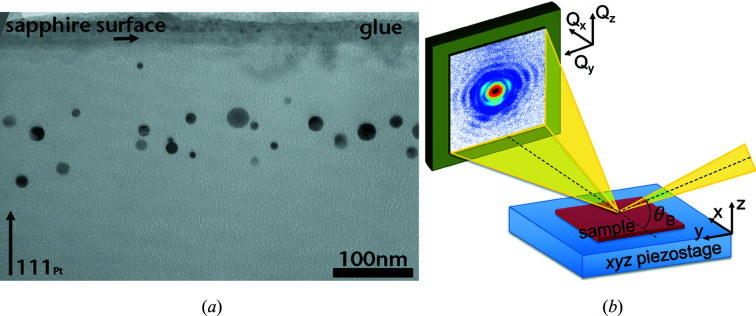
(*a*) Transmission electron microscopy image of a *c*(0001)_α_ substrate implanted with 1 × 10^16^ Pt^+^ cm^−2^ at room temperature and after annealing at 1873 K for 100 h. The [0001]_α_ crystallographic direction points vertically. (*b*) A scheme of the experimental setup.

The sample consists of Pt nanoparticles embedded in an α-alumina substrate (Santala *et al.*, 2011 ▸; Clauser *et al.*, 2020 ▸). The Pt particles were obtained by Pt implantation (Pt dose of 1.2 × 10^16^ atoms cm^−2^) at 600 keV at room temperature. The sample was then annealed at 1873 K for 10 h. After the annealing process, the cooling rate was 5 K min^−1^. This leads to Pt particles with different orientations within α-alumina, though the orientations are not completely random. Fig. 1 ▸(*a*) displays a transmission electron microscope image of the Pt particles for a sample obtained with conditions very close to those of the measured sample. The Pt diameter ranges from a few nanometres up to 50 nm.

The experiment was performed at the ID01 beamline (Leake *et al.*, 2019 ▸) with the EBS of ESRF. The BCDI experiment was performed at two beam energies: 9 keV (wavelength of 1.38 Å) and 10.3 keV (wavelength of 1.2 Å). The beam size was focused down to 73 (56) nm (vertically) × 73 (56) nm (horizontally) at 9 (10.3) keV using a tungsten or gold Fresnel zone plate (Leake *et al.*, 2017 ▸). The sample was mounted directly on an (*x* *y* *z*) scanning piezoelectric stage at 9 keV [see Fig. 1 ▸(*b*)] and on a mini goniometer mounted on the piezoelectric stage at 10.3 keV. The flux density delivered to the sample was 1.5 × 10^12^ and 6.4 × 10^12^ photons s^−1^ µm^−2^, respectively. The scanning stage has a stroke of 100 along *x* and *y*, with an encoder resolution of ∼1 nm. The diffracted beam was recorded with a two-dimensional Maxipix photon-counting detector (516 × 516 pixels with a pixel size of 55 × 55; Ponchut *et al.*, 2011 ▸) positioned on the detector arm at a distance of 0.13, 0.26 or 0.48 m at 9 keV and at 0.22 m at 10.3 keV. At 9 (10.3) keV, we measured the 111 Pt Bragg reflection in three dimensions by rotating the particle around its 111 Bragg angle through 3 (3.5)° in steps of 0.04 (0.1)°. We selected slightly misoriented particles [*i.e.* their (111) planes are slightly tilted (∼2–4°) with respect to the (0001) planes of the substrate], such that the Bragg peak and crystal truncation rod from the substrate (α-alumina) would not interfere with the signal scattered by the Pt particle.

## 3. Results and discussion

**Figure 2:**
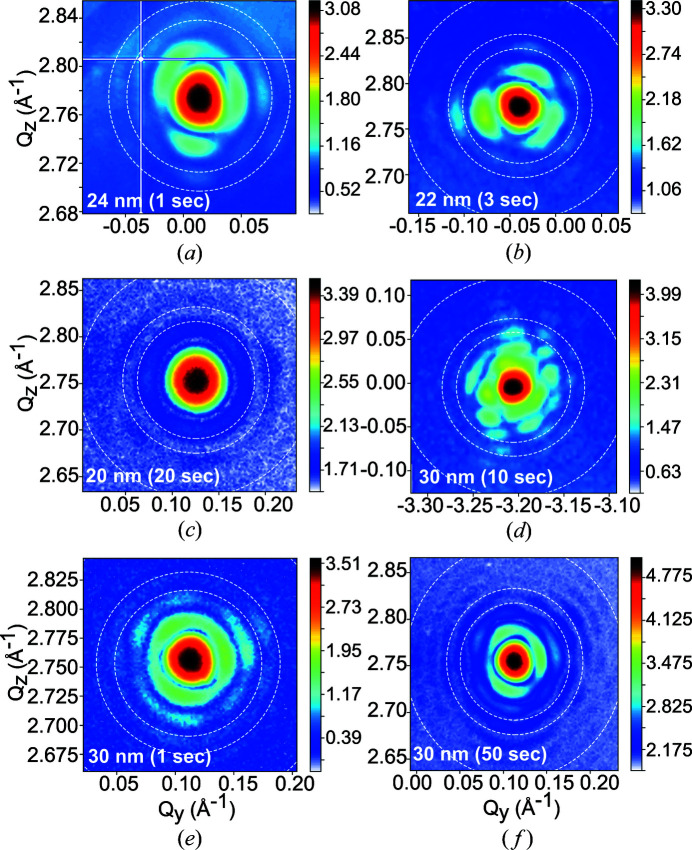
Two-dimensional detector images of the 111 Pt Bragg reflection of five measured Pt particles with a diameter of (*a*) 24 nm (*E* = 9 keV and a detector distance of 0.48 m), (*b*) 22 nm (*E* = 10.3 keV and a detector distance of 0.22 m), (*c*) 20 nm (*E* = 9 keV and a detector distance of 0.26 m), (*d*) 30 nm (*E* = 9 keV and a detector distance of 0.13 m; here the sample has been mounted vertically) and (*e*), (*f*) 30 nm (identical particle, *E* = 9 keV and a detector distance of 0.26 m), as a function of the *y* and *z* coordinates of the scattering vector **Q** (*Q*_*x*_ being along the beam direction) of the measured reflection. The intensity is displayed in logarithmic scale. The counting time is indicated as an inset in the figures. In (*a*), the white lines correspond to the gaps of the detector. The dashed white circles are single-pixel resolution shells corresponding to 10, 8 and 5 (not always visible) nm.

Fig. 2 ▸ displays two-dimensional detector images of five measured Pt particles as a function of the *y* and *z* coordinates of the scattering vector **Q** (*Q_*x*_* being along the beam direction) of the 111 Pt Bragg reflection. The intensity is displayed in logarithmic scale and indicates the number of counts per second. The size of the measured particles ranges from 20 to 30 nm. It has been estimated from the distance between thickness fringes in the detector plane. The interference fringes are a sensitive probe of changes in shape, size and strain (asymmetric intensity distribution of the diffraction pattern) of the investigated particle. In the case of a low-strain particle, from the periodicity of the fringes one can extract the particle size. In Fig. 2 ▸, the white circles are single-pixel resolution shells corresponding to 10, 8 and 5 (not always visible) nm. They give an estimation of the spatial resolution for the measurements. For long counting times (50 s), a spatial resolution of 5 nm can be reached for a particle with a diameter of 30 nm.

**Figure 3:**
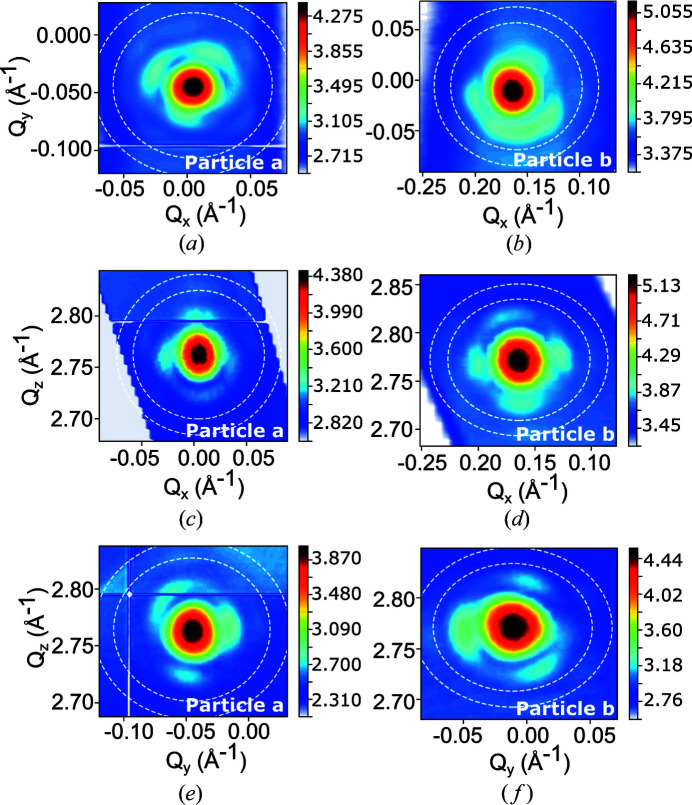
Three-dimensional reciprocal-space maps around the 111 Pt Bragg reflection as a function of the scattering vector coordinates (*Q*_*x*_, *Q*_*y*_ and *Q*_*z*_) for two measured Pt particles with a diameter of 24 nm [(*a*), (*c*) and (*e*)] and 22 nm [(*b*), (*d*) and (*f*)], corresponding to particle *a* and particle *b*, respectively [(*a*) and (*b*) in Fig. 2 ▸]. Single-pixel resolution shells corresponding to 10 and 8 nm are displayed. The counting time per detector frame was 1 s for particle *a* and 5 s for particle *b*.

As the Pt particles were embedded into α-alumina, they were stable under the beam. It was then possible to rock (rotate) the sample to measure the three-dimensional intensity distribution local to the Bragg peak of isolated Pt particles. Due to the sphere of confusion being larger than the beam size, it was necessary to realign the sample at each step of the rocking curve. Fig. 3 ▸ displays orthogonal cut planes through the three-dimensional diffraction patterns around the 111 Pt Bragg reflection of two of the measured particles with diameters of 24 nm (named, hereafter, particle *a*) and 22 nm (particle *b*). Before phase retrieval, we orthogonalized the data set using *xrayutilities*, a Python package, to take into account the curvature of the Ewald sphere (Kriegner *et al.*, 2013 ▸). The ‘fuzzy gridding’ function of *xrayutilities* was used to mitigate the possible loss of resolution (Kriegner *et al.*, 2015 ▸). Recent work has demonstrated a way to compute the point-to-point strain directly on a non-orthogonal grid (Maddali *et al.*, 2020 ▸). A series of 1000 relaxed averaged alternating reflections (RAAR; Luke, 2004 ▸) plus 2000 error reduction (ER; Gerchberg & Saxton, 1972 ▸; Fienup, 1978 ▸) steps, including a shrink-wrap algorithm every 50 iterations (Marchesini *et al.*, 2003 ▸), were used. The phasing process included a partial-coherence algorithm to account for the partially incoherent incoming wavefront (Clark *et al.*, 2012 ▸). To ensure the best reconstruction possible, we selected only the best three solutions (with lowest free log likelihood; Favre-Nicolin *et al.*, 2020 ▸) out of 30 with random phase start. The final solution is then obtained through an eigen decomposition of the best solutions and corresponds to the first mode of this decomposition (Favre-Nicolin *et al.*, 2020 ▸).

**Figure 4:**
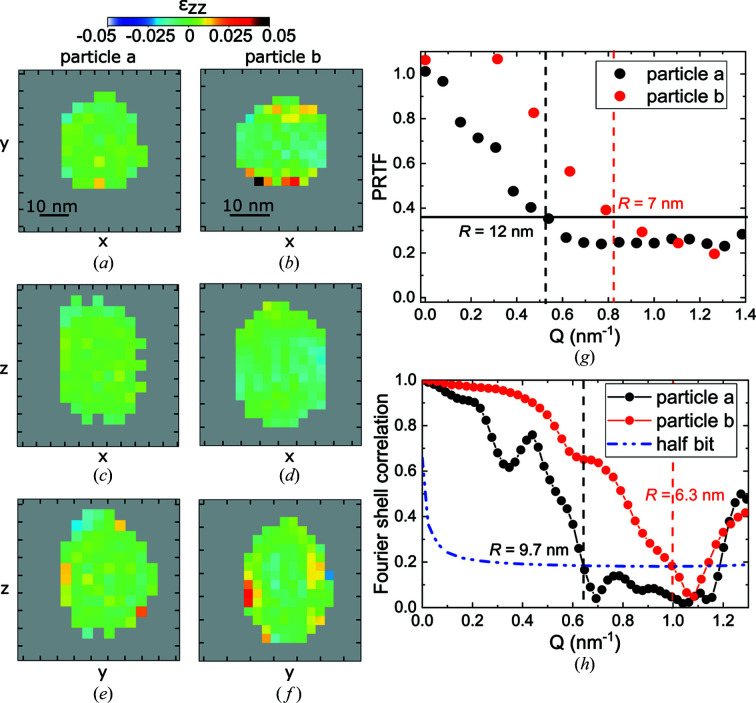
(*a*)–(*f*) Reconstructed out-of-plane strain (ε_*zz*_) of the two measured particles displayed in Fig. 3 ▸. Reconstructions in the *xy*, *xz* and *yz* planes. Tick spacing corresponds to 5 nm. (*g*), (*h*) Estimation of the spatial resolution using the PRTF and Fourier shell correlation for the two reconstructed particles *a* and *b*.

The reconstruction was then corrected for absorption, phase ramp and phase offset using the *bcdi* package (Carnis, Atlan *et al.*, 2021 ▸). A median filter with a 3^3^ window size was applied to the recovered phase, from which was derived the out-of-plane strain (ε_*zz*_ corresponding to ε_111_). Fig. 4 ▸ displays two-dimensional orthogonal slices of the reconstructed out-of-plane strain in the *xy*, *xz* and *yz* planes of the measured Pt particles whose reciprocal-space diffraction patterns are displayed in Fig. 3 ▸. The voxel size was 3.0 × 3.2 × 3.8 nm for particle *a* and 1.9 × 1.9 × 2.4 nm for particle *b*. Larger strain is observed at the particle interface with the sapphire matrix. To assess the quality of the reconstructions, the spatial resolution of the reconstructed particle has been evaluated using the phase retrieval transfer function (PRTF) (Chapman *et al.*, 2006 ▸; Cherukara *et al.*, 2018 ▸). The cut-off value was fixed at 1/*e*. As shown by the PRTF curves in Fig. 4 ▸(*g*), the spatial resolution is ∼12 nm for particle *a* and 7 nm for particle *b*. This is the resolution averaged over the three dimensions. The PRTF gives an upper limit of the estimated value of the spatial resolution averaged in the three directions.

We also used the Fourier shell correlation approach to estimate a lower bound to the three-dimensional resolution (van Heel & Schatz, 2005 ▸). To this aim, the full data set was separated into two parts. These two data sets were used to produce two reconstructions, from which the three-dimensional Fourier shell correlation curves were calculated [see Fig. 4 ▸(*h*)]. The decay of the curves below a 1/2 bit information threshold was used to define the cut-off, from which the spatial resolution was derived. The overall resolution was estimated as 9.7 nm for particle *a* and 6.3 nm for particle *b*, slightly better values than those given by the PRTF. For the measurements, a counting time of 1 or 5 s was used for particles *a* or *b*. An increase of the counting time improves the obtained spatial resolution (Cherukara *et al.*, 2018 ▸), as observed in Fig. 2 ▸. However, the *Q* ^−4^ decay of scattering intensity away from the Bragg peak requires a significant increase in exposure for resolution gain. For the measurements shown in Figs. 2 ▸ and 3 ▸, the oversampling was excessive. From a fringe-counting argument, to obtain a resolution of 2 nm on a 30 nm object, 15 fringes need to be measured; hence at an ideal oversampling ratio of 3, only 45 frames would be sufficient for the full rocking curve. If we expose at each frame for 50 s as shown in Fig. 2 ▸(*f*), a spatial resolution of 5 nm is readily achieved. In combination with more efficient focusing optics, such as Kirkpatrick–Baez mirrors or multilayer Laue lenses, in conjunction with a multilayer monochromator which fully exploits the coherence of the incoming light (Leake *et al.*, 2019 ▸), one would anticipate to be able to obtain an additional factor of 80–100 (4–5 in the focusing optics and 20 in the monochromator). In the case of strongly scattering materials, such as Pt, a resolution of 2 nm on a 30 nm object would be feasible in a few minutes.

## 4. Conclusions

In summary, we have demonstrated the possibility of measuring single nanoparticles smaller than 30 nm by using Bragg coherent diffraction imaging. Their structure (three-dimensional shape or strain fields) can be recovered. As synchrotron machines evolve (recent and upcoming upgrades of worldwide X-ray light sources) and the X-ray optics used to exploit the sources improve (*i.e.* improvement of focusing optics and use of transfocators; Vaughan *et al.*, 2011 ▸), the coherent flux density on samples will continue to increase. As things stand today, a factor of 100 improved flux density is readily achievable, which allows the imaging of smaller/similar particles with better spatial and time resolutions. This work showcases the possibility of imaging three-dimensional and *in situ* nanoparticles smaller than 30 nm, assuming their stability. These measurements will prove invaluable for future nanomaterial optimization and design.
